# Plasma-Activated Water for Food Safety and Quality: A Review of Recent Developments

**DOI:** 10.3390/ijerph19116630

**Published:** 2022-05-29

**Authors:** Mizanur Rahman, Md. Shariful Hasan, Raihanul Islam, Rahmatuzzaman Rana, ASM Sayem, Md. Abdullah As Sad, Abdul Matin, António Raposo, Renata Puppin Zandonadi, Heesup Han, Antonio Ariza-Montes, Alejandro Vega-Muñoz, Atiqur Rahman Sunny

**Affiliations:** 1 Department of Food Engineering and Tea Technology, Shahjalal University of Science and Technology, Sylhet 3100, Bangladesh; mizan-fet@sust.edu (M.R.); sharifulh.nahid@gmail.com (M.S.H.); raihanul08@student.sust.edu (R.I.); rzaman-fet@sust.edu (R.R.); asm.sayem-fet@sust.edu (A.S.); 2 Department of Food Engineering, N P I University of Bangladesh, Manikganj 1800, Bangladesh; abdullahfet7@gmail.com; 3 Department of Food Processing and Engineering, Chattogram Veterinary and Animal Sciences University, Chattogram 4225, Bangladesh; abmatin@cvasu.ac.bd; 4 CBIOS (Research Center for Biosciences and Health Technologies), Universidade Lusófona de Humanidades e Tecnologias, Campo Grande 376, 1749-024 Lisboa, Portugal; 5 Department of Nutrition, Campus Darcy Ribeiro, University of Brasilia, Asa Norte, Distrito Federal, Brasilia 70910-900, Brazil; renatapz@unb.br; 6 College of Hospitality and Tourism Management, Sejong University, 98 Gunja-Dong, Gwanjin-Gu, Seoul 143-747, Korea; 7 Social Matters Research Group, Universidad Loyola Andalucía, C/Escritor Castilla Aguayo, 4, 14004 Cordoba, Spain; ariza@uloyola.es; 8 Public Policy Observatory, Universidad Autónoma de Chile, Santiago 7500912, Chile; alejandro.vega@uautonoma.cl; 9 Department of Genetic Engineering and Biotechnology, Shahjalal University of Science and Technology, Sylhet 3100, Bangladesh; a.rsunny@cgiar.org or; 10 Suchana Project, WorldFish, Bangladesh Office, Gulshan, Dhaka 1213, Bangladesh

**Keywords:** food quality, microbial inactivation, plasma-activated water (PAW), non-thermal plasma, physicochemical properties

## Abstract

Plasma-activated water (PAW) has received a lot of attention lately because of its antibacterial efficacy and eco-friendly nature. Compared to traditional disinfectants, this novel and intriguing option has a high disinfectant capacity while causing little to no modifications to the foodstuffs. Until now, PAW has successfully demonstrated its effectiveness against a broad range of microorganisms on a wide variety of food items. Though the efficacy of PAW in microbial reduction has been extensively reviewed, a relatively significant issue of food quality has been largely overlooked. This review aims to summarize the current studies on the physicochemical characteristics and antimicrobial potential of PAW, with an in-depth focus on food quality and safety. According to recent studies, PAW can be a potential microbial disinfectant that extends the shelf life of various food products, such as meat and fish products, fruits and vegetables, cereal products, etc. However, the efficacy varies with treatment conditions and the food ingredients applied. There is a mixed opinion about the effect of PAW on food quality. Based on the available literature, it can be concluded that there has been no substantial change in the biochemical properties of most of the tested food products. However, some fruits and vegetables had a higher value for the enzyme superoxide dismutase (SOD) after PAW treatment, while only a few demonstrated a decrease in the Thiobarbituric acid reactive substances (TBARS) value. Sensory properties also showed no significant difference, with some exceptions in meat and fish products.

## 1. Introduction

Food consumption is rising in tandem with the world population. The most challenging task is to produce safe, high-quality food while considering the new hazards that emerge as a result of developing pathogens during food production. Until today, foodborne illness caused by contaminated food continues to be a major concern [1]. Consumption of contaminated foods or beverages can result in outbreaks of foodborne diseases. According to Song et al. [2], these outbreaks are anticipated to increase in the future as a result of growth in numerous businesses, a growing global population, and the implications of environmental pollution. Therefore, food scientists have the responsibility to maintain food safety and quality.

To cope with the problem, scientists are trying to develop a method to reduce contamination while also improving the food’s shelf life and nutritional value. Chemicals, refrigeration, and thermal control are all frequently used methods for contamination control. These techniques, however, may have an adverse effect on the physical, biochemical, or sensory characteristics of foods [3]. Chemicals used in decontamination, such as chlorine dioxide, organic acids, and dense phase CO_2_, also have a negative effect. Chlorine may combine with organic materials to produce trihalomethanes, a possible carcinogen that may be harmful to human health [4]. Thermal processing can result in textural damage, alterations in flavor and sensory qualities, and nutritional value loss [5,6]. In recent years, numerous non-thermal food processing systems have been extensively investigated to reduce these negative effects, such as high hydrostatic pressure [7,8], pulsed electric field [9], ultrasound [10], irradiation [11,12], and non-thermal plasma [13,14,15]. Although Food Irradiation has been approved by the Food and Drug Administration (FDA), World Health Organization (WHO), U.S. Department of Agriculture (USDA), and other national and international agencies, the safety of irradiated food may prompt a scope of questions. The physical properties and the functionality of pectin, gums, cellulose, and starch are affected by irradiation; hydroperoxides are produced from lipids; oxidation of myoglobin by radiolytic products causes discoloration of meat and fish products [16]. Properly irradiated food products are microorganism-free, but it does not eliminate toxins produced by them [17]. However, non-thermal plasma or cold plasma has attracted a lot of attention, as it has shown great potential against a wide variety of microbes [18,19,20]. An atmospheric pressure plasma jet (APPJ) and a dielectric barrier discharge (DBD) are two of the common devices used in this technology, both of which have limited wide-scale uses due to their inability to expose vast food surface areas and volumes. Moreover, food products’ highly irregular surface topography provides numerous hiding places for microorganisms, enhancing their resistance to cold plasma treatment. Plasma-activated water (PAW) was developed to resolve this issue, as it can treat the entire surface of the food on a broader scale. It is also an eco-friendly and cost-effective disinfectant with exceptional and broad antibacterial activity, opening up new application possibilities in the food, agricultural, and biomedical industries [21,22].

When non-thermal atmospheric plasma reacts with water, the result is PAW. It contains a diverse range of highly reactive oxygen and nitrogen species (RONS) [23,24]. The chemical and biological effects of PAW are attributed to these reactive species. Depending on the storage conditions, the reactivity and antibacterial characteristics of PAW can be maintained for long periods. Since the initial successful research of PAW’s antibacterial activity in fresh strawberries [25], numerous studies have been conducted to evaluate its effectiveness against other food items. Recent review articles have focused exclusively on PAW generation, its physicochemical properties, microbial inactivation mechanisms, their reactive chemistries, in vitro biological activity, and agricultural and medical uses [26,27,28]. Few reviews have focused on food safety and quality parameters resulting from PAW treatment. Thus, the current review aims to represent the uses of PAW for food decontamination and their impacts on food quality, the factors affecting the efficiency of PAW, and its use in conjunction with other technologies as documented in recent studies.

## 2. PAW Generation

**Figure 1:**
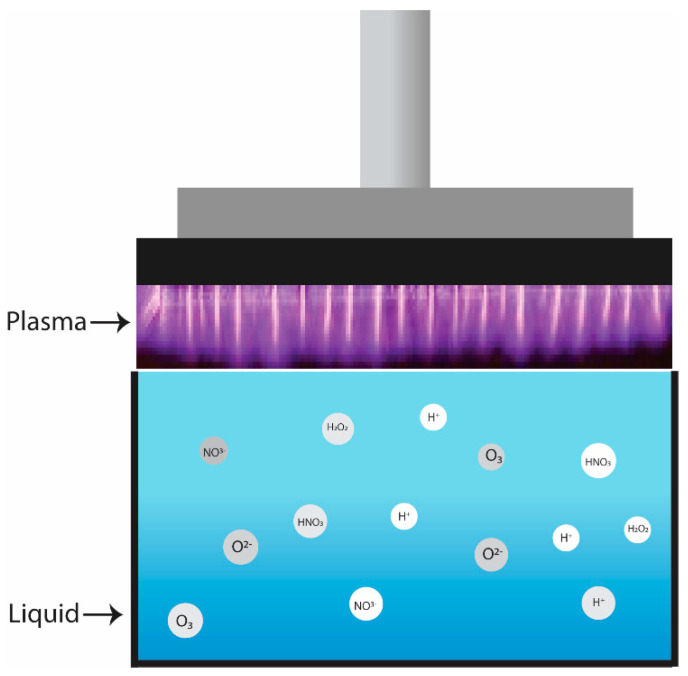
Plasma-activated water generation.

PAW is generated by water sample and non-thermal plasma (NTP) active particle interactions (shown in Figure 1) [25,29]. Active molecules and electrons are created when ambient air is brought into the plasma phase [30,31]. Water molecules interact with these plasma-generated reactive particles to produce PAW [29]. Several NTP–water reaction systems, such as dielectric barrier discharge (DBD), pulsed corona discharge, atmospheric pressure plasma jet (APPJ), and gliding arc discharge, are reportedly used to generate PAW in rice cake [32], protein extractions [33], eggs [34], and chicken breast [35], respectively. However, due to their ease of use and capacity to produce highly reactive species in PAW, DBD and APPJ are the most broadly employed [36]. In DBD plasma, a large capacitance circuit is used to oscillate a current across the plasma to stimulate it in a natural breakdown manner [19], whereas a jet nozzle with a lower diameter is used in APPJ plasma. Scientists have used APPJ in many food products, such as bean curd [37], tomato surface [38], Chinese bayberry [39], etc., and DBD on cooked chicken surface [40], button mushrooms [41], shiitake mushroom [42], etc. The types and concentrations of reactive species found in PAW are influenced by the gases and liquids used to create the plasma [43]. In several studies, PAW was made with sterile distilled water (SDW), tap water (TW), reverse osmosis water (ROW), and deionized water (DIW). Among these, SDW and DIW were the most effective at deactivating microbes in in vitro studies [34,44,45]. DIW with 1% sodium pyrophosphate (SPP) is often used as a curing agent in PAW to prevent NO2 concentration [46,47,48]. Bolouki et al. [49], in their study, stated that air and nitrogen rapidly decreased the pH of PAW. Meanwhile, using argon and oxygen, the pH remained relatively stable.

## 3. Physicochemical Properties of PAW

When plasma interacts with liquids, several complex chemical reactions occur at the interface between the two media, creating reactive species and altering the physicochemical properties of the treated solutions, such as electrical conductivity, oxidation–reduction potential (ORP), and pH.

### 3.1. Chemical Properties of PAW

Various events occur in the aqueous solution during PAW formation, including gaseous species transfer, chemical interactions between gaseous species, and liquid molecules [24]. Because of this, a number of reactive species are produced. Bruggeman et al. [50] covered this subject thoroughly in a review. Plasma treatment causes nonequilibrium dissociation of water molecules, resulting in the creation of short-lived species, including hydrated electrons and hydroxyl ions (OH^−^) [23], which quickly react to create stable species, such as ozone (O_3_), superoxides (O^2−^), and hydrogen peroxide (H_2_O_2_). Hydroxyl radicals (OH•) are extremely reactive and short-lived species with extremely high redox potential that interact with others in the liquid to produce new radicals, hence initiating additional reactions. H_2_O_2_, on the other hand, is a rather stable species. It is a physiologically active agent with strong antibacterial and cytotoxic effects [51]. Along with reactive oxygen species (ROS), PAW also contains reactive nitrogen species (RNS), such as nitric oxide NO, peroxynitrate O_2_NOO^−^, peroxynitrite ONOO, and nitrogen dioxide (NO_2_•) radicals. RNS are made when a non-thermal plasma comes in contact with a liquid. This is caused by a number of reactions that happen in the gas, in the liquid, and at the interface between the gas and liquid, all of which contribute to the complex environment in PAW in varying degrees [52,53]. The detection of these species in liquids is difficult and usually requires the use of indirect traces.

### 3.2. Physical Properties of PAW

When generating PAW, the reaction between plasma species and water causes an acidification of the water, which is indicated by pH. The production of strong acids lowers the pH of PAW as treatment time increases [29,54]. Nitric and nitrous acid formation is thought to be the primary cause of the pH drop [55]. The pH level and the generation of acidified compounds are varied based on the discharge method, type of plasma used, and the gases fed into the system [56]. The polarity of the applied voltage also influences the pH, with negative discharge causing a higher decrease than positive [52]. According to Pemen et al. [57], a regulated pH can be achieved by combining thermal and non-thermal plasmas.

The oxidation–reduction potential (ORP) is a property of solutions that indicates their ability to oxidize or reduce another material. ORP is considered a key component in the inactivation of microorganisms since it destroys the cell membrane and the cellular defense mechanisms of the microbes [58]. H_2_O_2_ is considered to be the most important ROS produced in PAW, as it is primarily involved in redox processes [52]. According to different studies, treatment time [25] and mode of generation [25] affect the ORP value of PAW. Notably, PAW generated above the water’s surface had a greater ORP value (up to 20%) than PAW generated under the water’s surface [25]. However, the ORP values of PAW did not vary much when stored at different temperatures, but they did decrease when stored at varied periods [29].

Electrical conductivity is a property of aqueous solutions that indicates their ability to conduct electricity, which is dependent on the concentration of charged species and ions. The presence of increased ROS and RNS in plasma-activated water positively affects the conductivity of PAW. According to Wu et al. [59] and Ma et al. [25], the electrical conductivity of PAW considerably increased as the activation time increased. However, it was inversely related to pH. The electrical conductivity of a solution increases as the pH decreases because of the greater mobility of H^+^ ions than OH ions [52]. Furthermore, PAW produced below the water’s surface has a greater conductivity than PAW produced above [54].

## 4. PAW for Food Safety

Food safety is one of the most pressing issues in the modern world. Unsafe food containing pathogenic bacteria, viruses, parasites, or chemical compounds can cause disorders ranging from diarrhea to cancer [60]. To manage both microbiological and chemical dangers, new food processing methods, such as non-thermal technologies, have been tested during the past two decades. However, these technologies have drawbacks, such as altering the physical and chemical properties of treated foods [61]. Non-thermal plasma (NTP), a cutting-edge technique capable of removing both biological and chemical pollutants, has lately attracted the attention of several researchers for its outstanding results [26].

### 4.1. Antimicrobial Mechanisms of PAW

Several studies have been conducted to find out the antibacterial properties of PAW since Kolikov et al. [62] published the first in vitro study of PAW. However, there is still disagreement about the most effective inactivation agents because of their harder identification. According to Zhang et al. [63], ROS such as hydroxyl radical (OH•), H_2_O_2_, and ozone (O_3_) were crucial in the inactivation of *S. aureus*. Meanwhile, O_3_ and H_2_O_2_ were proposed to be the main cause according to Thirumdas et al. [22]. Because they are easy to identify, long-lived reactive oxygen and nitrogen species (RONS), including NO_2_^−^, H_2_O_2_, NO_3_^−^, and O_3_, are described as the main cause of bacterial inactivation [4,64]. The acidic pH of the solution also helps in the process.

**Figure 2:**
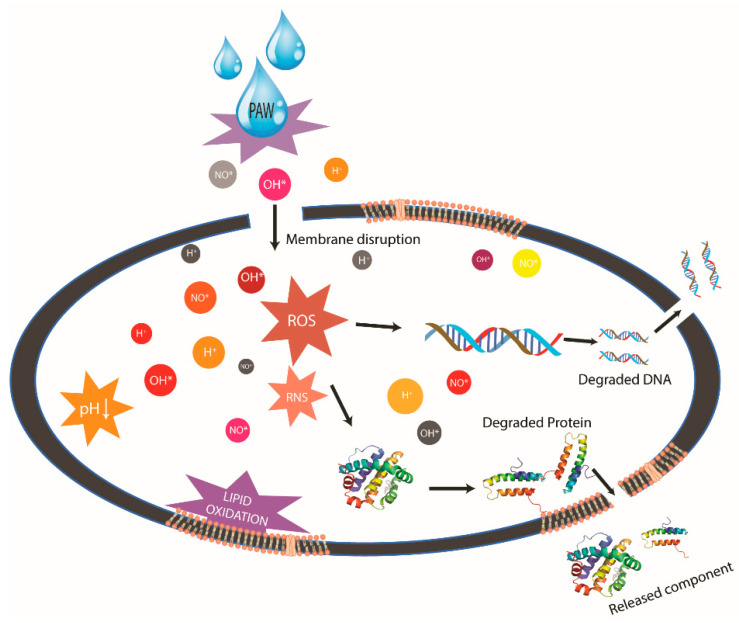
Antimicrobial mechanisms of PAW.

After being exposed to PAW, membrane disruption of the microbial cell and degradation of intracellular contents have been observed [20]. RONS in PAW can induce cell membrane rupture due to lipid peroxidation resulting from oxidative stress [63]. Moreover, PAW has been shown to generate extremely high-intensity electric fields [52], which increases membrane permeability and creates temporary pores allowing reactive species into cells [22]. When reactive species infiltrate cells, they interact and degrade the internal organs, including proteins, DNA/RNA, ribosomes, and mitochondria [65]. Then, the degraded intracellular components (proteins and nucleic acids) are released (shown in Figure 2) [54,66]. Additionally, it has been proposed that water molecules may potentially enter cells following membrane leakage, causing swelling that leads to cell death [30].

In addition to the bacterial decontamination process, in the food manufacturing industry, fungi-infected parts of food or mycotoxin are removed using a variety of physical and chemical detoxification and decontamination techniques. There are a number of variables that influence the effectiveness of PAW treatments in removing mycotoxins from food, including the plasma device, the treatment parameters (such as power supply, feed gas type, and treatment duration), the species of fungus, the type of mycotoxin, and the food matrix itself [67]. According to some recent studies, the degradation of the aflatoxin AFB1 is triggered by long-lived ROS, which break down the toxic site C8 = C9 double bonds in the furan rings [68,69].

### 4.2. Chemical Decontamination with PAW

The continuous and indiscriminate use of agrochemicals has many negative impacts on humans, the environment, and biodiversity. PAW has a revolutionary use in degrading pesticides and other chemical contaminants on the surface of food. A significant amount of chemical degradation from food surfaces was reported in various studies after PAW treatment; for instance, 73.60%, 51.97%, and 34.6% residual reductions were reported from grapes [70], tomatoes [71], and barley [72], respectively. However, the toxicological effect of the metabolites of degradation on food surfaces is still a concern. That is why it remains a great opportunity for future investigation.

## 5. Application of PAW in Food

In recent years, there have been many studies showing the effectiveness of PAW as a food decontaminant for both agricultural and animal-derived products. In those studies, many fruits and vegetables, such as strawberries, tomatoes, grapes, and Chinese blueberries, along with various meat and meat products, such as chicken breast, fresh beef, and shrimp, have been tested. These studies reveal that PAW could be applied to inactivate microorganisms with little or no damage to the food products shown in Table 1. According to reports, several crucial characteristics, such as food surface texture [73], treatment duration [31,44,74], plasma power during PAW formation [34], and organic matter [75], were shown to influence the effectiveness of microbial inactivation on foods. In order to keep harmful germs in check and food quality high at the same time, these variables must be optimized [34,44,76].

### 5.1. Decontamination of Agricultural Products

The main barrier to handling agricultural products is their great susceptibility to postharvest spoiling, with the rapid deterioration of quality characteristics [82,83]. For the production and distribution of food in a sustainable manner, PAW is proving to be very useful. Decontamination utilizing PAW has recently been successfully used on the surface of a wide range of foods as a result of successful in vitro studies.

#### 5.1.1. Fruits and Vegetables

PAW was initially investigated on fruit by Ma et al. [25], where PAW was applied to *Staphylococcus aureus* inoculated strawberries, which showed a reduction of 1.7–3.4 log CFU/g when stored for 4 days [25]. A similar experiment was performed on Chinese bayberries to reduce the effect of both bacteria and fungi, which showed around 1.1 log CFU/g reduction when treated by PAW and stored for 8 days [39]. The study also revealed that PAW treatment reduced fruit decay by about 50% compared to control [39]. Later, Guo et al. [73] applied PAW on grapes with the activation time varied between 30 and 60 min and found 0.38–0.53-log CFU/mL reduction in yeast without affecting the quality of the grape. In a recent study, Hou et al. [38] found that using two plasma jets to produce PAW instead of one increased the bacterial reduction on the tomato’s surface. The study reported more than 5 log reductions of *E. coli*, L. monocytogenes, and *S. Typhimurium* bacteria when treated for 210, 30, and 180 s, respectively. On tomatoes, PAW reduced the thiram (THM) and chlorothalonil (CTL) residue to 65.89 and 75.07% [84]. The reduction rate was found to be higher with the increasing PAW exposure time.

The effects of PAW rinsing on baby spinach leaves for microbial inactivation were observed by Risa vaka et al. [80], which showed a total bacterial reduction of about 1 log CFU/g. In another study, PAW treatment for 3 min on lettuce showed a decrease in *Pseudomonas fluorescens* bacteria below detection level. In comparison, around 2.4 log CFU/g reduction was seen for *Listeria innocua* when treated for 5 min [85]. Recently, a study on rocket leaves showed around 1.7–3 log CFU/g reduction in total psychotropic and mesophilic bacteria when washed with PAW for 2–5 min [77]. Some studies showed the microbiological effectiveness of subsequent mild heat along with PAW [44,86]. In a study on salted Chinese cabbage, mild heat of 60 °C was applied in addition to PAW, which showed improved microbial reduction compared with PAW alone [44]. Later, Xiang et al. [86] showed a similar result in their study.

Since fresh-cut fruits and vegetables are readily available, reasonable, and easy to access, people are leaning towards them. However, due to their perishable nature and minimal processing, the risk of contamination is high. To reduce the contamination and enhance shelf-life, PAW treatment can be applied to fresh-cut products. For instance, Liu et al. [87] showed that fresh-cut apples immersed in PAW for 5 min effectively reduced the microbial activity on day 12. PAW generated at 7.0 kHz with 8 kV amplitudes showed the most efficiency, reducing 1.05 log CFU/g of aerobic bacteria. Coliforms, molds, and yeasts were reduced to 0.86 log CFU/g, 1.04 log CFU/g, and 0.64 log CFU/g, respectively. Using fresh-cut pears also showed comparable results [88]. Recently, a study conducted on ready-to-eat rocket salad demonstrated a reduction of 1.58 log CFU/g in total microbial activity, increasing the shelf life from 3 to 7.5 days (~2.5 times) while retaining the quality [89].

#### 5.1.2. Other Agricultural Products

Several studies looked into the use of PAW on edible mushrooms, such as button mushrooms and shiitake mushrooms. A study conducted by Xu et al. [31] found that maintaining button mushrooms for 7 days at 20 °C while immersed in PAW reduced the fungal and bacterial counts by 0.5 log and 1.5 log CFU/g, respectively, which is similar to the result of a recent study conducted by Zhao et al. [41]. In another survey of shiitake mushrooms, after PAW treatment, the total bacterial count was reduced to 0.89 log CFU/g while decreasing the overall color changes [42]. The studies show that PAW application is very effective for microbial decontamination in edible mushrooms while better retaining the physical characteristics.

### 5.2. Decontamination of Animal-Derived Products

Along with fruits and vegetables, animal-derived foods such as meat, fish, and eggs are also included in our balanced diet. Due to being prone to microbial attack, the quality of animal-derived foods degrades more rapidly. Scientists have conducted many studies to reduce this microbial contamination and produce safe and quality products [4,90,91]

#### 5.2.1. Fish and Fish Products

Because seafood is transported at large distances, it requires a longer shelf life. However, due to the microbial activity, the quality of the product degrades rapidly. In a study, Liao et al. [92] used PAW ice instead of tap water to store shrimp. The results demonstrated a substantial reduction in microbial growth, allowing the product to be kept fresh for an additional 4–8 days. The deterioration of color and hardness was also slowed down by the PAW ice treatment. Recently, *P. fluorescens* cultured on fresh mackerel fillets was reduced by 0.4 log after PAW treatment [93]. Later, Zhao et al. [91] discussed the combined effect of PAW with other technologies. PAW application on grass carp also showed promising results by reducing *S. Typhimurium* and *L. monocytogenes* up to 1.44 and 1.21 log. Another experiment on the Yellow River carp fillets showed PAW effectiveness against *Shewanella putrefaciens* bacteria by reducing 1.03 log CFU/g [78]. Although the lightness value (L*) of the fillets increased, the redness value (a*) significantly declined, but the yellowness value (b*) remained unchanged compared to sterile deionized water treatment. The positive thing is that it caused no significant change in the sensory and textural properties [78].

#### 5.2.2. Meat and Meat Products

Zhao et al. [90] researched the bacterial inactivation of fresh beef using PAW. The result demonstrated roughly 3.1 log CFU/g reduction in bacteria when treated for 24 h, which extended the shelf life for around 4–6 days. Moreover, it showed that treatment intervals and storage time could influence the efficacy of PAW on fresh beef. In another research, Qian et al. [94] demonstrated PAW treatment to reduce around 1 log CFU/g of *Salmonella* Enteritidis. Along with beef, PAW treatment has also been tested with chicken [35,40,76]. Kang et al. [35] showed that, when treated for 12 min, PAW produced by gliding arc discharge plasma reduced *P. deceptionensis* CM2 to about 1.05 log CFU/g on chicken breast. However, these findings were lower than those of a previous in vitro study that found a 5 log CFU/mL reduction after 10 min of PAW treatment [20]. In another study on chicken skin, PAW treatment for 60 min reduced *E. coli* K12 and *S. aureus* by 0.46 log CFU/mL and 0.33 log CFU/mL, respectively [76]. Recently, PAW was applied to cooked chicken breasts by Wang et al. [40], where cooked chicken breasts were soaked in PAW for 20 min. The result showed a reduction of around 2.09 and 2.29 log CFU/g for methicillin-resistant *Staphylococcus aureus* bacteria and methicillin-susceptible *S. aureus* bacteria.

#### 5.2.3. Eggs

Lin et al. [34] looked into the antibacterial activity of PAW against *Salmonella* Enteritidis found in eggshells. They applied PAW for 60, 90, and 120 s, in which 120 s of treatment time showed the most reduction of 5.51 log CFU/egg, while 60 and 90 s showed a reduction of 4.41 log CFU/egg. They also stated that the removal of bacteria by PAW washing was three times higher than the water washing. In another study, after 60 s of treatment, *Salmonella* Enteritidis was reduced to 2.84 log CFU/egg from 7.92 log CFU/egg utilizing two plasma jets [4].

### 5.3. Application of PAW in Processed Foods

Along with other foods, PAW has been successfully implemented in microbial decontamination of processed food. In a study, Zhai et al. [37] assessed the antimicrobial activity of PAW on thin sheets of bean curd. After 30 min of PAW treatment, 1.26 log CFU/g reduction in total aerobic bacteria was seen. Total yeast and mold count was also reduced to 0.91 log CFU/g, which showed the potential application of PAW in the microbiological decontamination of processed foods. Later, Han et al. [32] experimented with PAW on a Korean rice cake, where PAW was generated using two atmospheric dielectric barrier discharges for 20 min. The result showed the decrease in total aerobes to 2.78 log CFU/g while retaining the color values and firmness. Similar results were also observed in PAW-treated tofu [95].

## 6. Impact of PAW on Food Quality

Food quality and safety are the major concerns in food production. Because PAW contains reactive oxygen and nitrogen species (RONS), a food’s biochemical and sensory characteristics can be affected in either a positive or negative way. These effects are summarized in Table 2.

### 6.1. Biochemical Properties

#### 6.1.1. Vitamin

Vitamins are unstable during processing treatment. Hence, it is essential to investigate the stability of vitamins during plasma-activated water treatment. There are few studies that have been conducted to determine vitamin stability following PAW treatment. Xu et al. [31] concluded that PAW had a favorable impact on vitamin C levels in button mushrooms throughout postharvest storage. A similar result was reported by Zheng et al. [70] and Xiang et al. [86]. During 12 days in storage, Liu et al. [87] discovered that vitamin C concentration in fresh-cut apples treated with PAW was reduced. However, compared to untreated apples, this constantly reducing tendency was not considerably significant. In summary, these studies demonstrate that the stability of vitamin C may not be much affected by PAW treatment. However, the topic should be further studied to make a clear statement.

#### 6.1.2. Antioxidant

Antioxidants are substances that can help prevent or delay cell damage caused by free radicals, or unstable molecules. They contribute to the preservation of nutrients and the sensory qualities of foods, such as color, texture, freshness, scent, and taste [97]. Antioxidant-rich foods may help to reduce the chance of developing a number of diseases, including heart disease and certain types of cancer [98,99]. Bioactive substances found in fresh fruit and vegetables have been shown to significantly impact human health, mostly due to their antioxidant qualities. Several relevant studies have shown that, after being washed with PAW, no changes were observed in the antioxidant activity of fresh-cut apples [87], pears [88], or button mushrooms [41]. In a recent study, slight changes in total flavonoid content was found when applying PAW [77], but no changes in total phenolic content were seen [77,84]. It was similar to the phenolic compound change in tomatoes [84]. In another study, Zhao et al. [41] determined the radical-scavenging activity of button mushrooms using DPPH tests. Compared to the control, it showed no noticeable difference in antioxidant activity. However, they found that the DPPH radical-scavenging activity reduced more rapidly as PAW discharge increased. A similar trend was seen in fresh-cut apples [87]. Since grapes and juice contain significant amounts of antioxidants, including phenolic compounds, anthocyanins, and flavonoids [100], when treated with PAW and mild heat, there was no substantial distinction between grapes in terms of 2, 2′-Azino-Bis-3-Ethylbenzothiazoline-6-Sulfonic Acid (ABTS) radical-scavenging capacity and fluorescence recovery after photobleaching (FRAP) value. Xiang et al. [101] also found similar results using PAW on mung bean sprouts.

#### 6.1.3. Protein

Proteins are important in metabolism. Besides providing the body with energy, they are the primary building blocks of its complex architecture. The effect of PAW on protein substances was observed in recent studies. Liao et al. [92] evaluated the total sulfhydryl group levels and Ca^2+^ ATPase activity in shrimps treated with PAW ice, as its activity shows the total myosin denaturation and damage, finding no significant change in those values relative to iced water treatment. Evaluation of myofibrillar proteins (MPs) is important, as the majority of total muscle proteins are myofibrillar proteins (MPs) [102]. Qian et al. [103], in their study, found a decrease in sulfhydryl groups content of MP extracted from chicken meat, which resulted in protein oxidation after PAW application. However, analyzing the myofibrillar protein in chicken drumsticks after PAW treatment, Qian et al. [104] found that reactive species in plasma-activated water did not degrade the proteins. Liao, et al. [105] observed a similar finding with beef. Applying ultrasound with PAW, the protein content of treated chicken flesh did not alter much [76]. In another study, plasma-activated lactic acid showed no significant changes in the secondary structure of the beef protein [94].

#### 6.1.4. Enzyme

Food enzymes have both favorable and unfavorable effects on food products. Some enzyme activities cause food quality to deteriorate rapidly, decreasing shelf life and requiring control methods. Few studies show the effect of PAW treatment in foods [41,70,96]. In a study on button mushrooms, Xu et al. [31] evaluated the effect of PAW on the superoxide dismutase (SOD) enzyme, as it is considered to be the primary enzymatic antioxidant involved in mushroom responses to stress factors. The result showed greater SOD content during storage after PAW treatment. Later studies on fresh-cut kiwifruits [96], grapes [70], and button mushrooms [41] showed a similar result. Zhao et al. [41] also measured the polyphenol oxidase (PPO) and peroxidase (POD) activities in button mushrooms after PAW treatment, as both cause postharvest browning of mushrooms. PAW treatment showed significantly lower activity of PPO and POD compared to water treatment, which was relevant to the study on salted Chinese cabbage [44]. In contrast, PAW treatment on fresh-cut kiwifruit found an increase in peroxidase (POD) and catalase (CAT) activity [96].

#### 6.1.5. Carbohydrate

Carbohydrates are made up of fiber, starches, and sugars. Among them, sugar is widely regarded as the primary carbohydrate found in food [106]. In fruits, such as grapes, no substantial changes in total soluble solids (TSS) and sugar were found when PAW was applied [70,86]. Similarly, Choi et al. [44] also demonstrated no significant change in sugar content on shredded salted Chinese cabbage. As there are few studies on the effect of PAW on sugar and other carbohydrates available, we cannot say anything with certainty. This should be a great area to work on in the future.

#### 6.1.6. Lipid

Lipid oxidation is a significant factor in quality degradation, affecting color, flavor, safety, and nutritional value. Lipid peroxidation may occur due to interactions between PAW carrying ROS and food cells [46]. Researchers are trying to find out iwhether PAW influences lipid oxidation. The primary and secondary lipid oxidation products are commonly determined using peroxide value (PV) and TBARS analyses [91]. In their study, Jung et al. [48] found the PV value to increase during 21 days of storage of PAW-cured sausage, but the value decreased compared to 21 days of storage when the storage time was extended to 28 days. Yong, Park, Kim, Jung, Park, Lee, Choe, Jo and Polymers [46] saw a similar trend in the PV value in loin ham when stored for two weeks. In a recent study, PAW + US treatment on mackerel fillets showed a PV value of 0.5 meq O_2_/kg lipid, where the maximum permissible value of peroxide in marine lipids is 5 meq O_2_/kg [107].

Zhao et al. [41] studied the change of malondialdehyde (MDA) content in button mushrooms after PAW treatment and saw a significant decrease compared to control when stored for 12 days. A similar observation was also seen in goji berries [108]. These findings showed the effectiveness of PAW in reducing membrane lipid peroxidation. Furthermore, Liao et al. [105] reported no significant change of TBA for beef during thawing with PAW, which agrees with the result of the TBARS value of PAW-cured dried pork loins [109]. In another study, Liao et al. [92] stored shrimp with PAW ice for 7 days. Analyzing the shrimps after the storage time showed a lower TBA value relative to tap water ice storage. They assumed that lower microbial growth due to PAW ice treatment led to lower lipid oxidation. Similarly, Luo et al. [109] observed that dried pork loins cured with PAW was characterized by a considerably decreased TBARS value, whereas no significant change in the TBARS value was seen in tiger nuts [110] or beef [90,94] after PAW treatment.

### 6.2. Sensory Properties

#### 6.2.1. Color

Food’s color is possibly the most essential sensory attribute. It is also the first thing that a consumer notices. Color has a significant role in consumers’ perception of a product’s freshness, flavor, and quality [111]. Any undesired color change in food products as a result of processing will be a major barrier to customer acceptance. There are numerous studies stating the effect of PAW on food color parameters [73,76,77].

In most agricultural products, such as grapes [73,86], fresh-cut endive lettuce [112], baby spinach leaves [80], fresh-cut potatoes [113], fresh-cut kiwifruit [96], fresh-cut apples [87], rice [114], strawberries [25], and mushrooms [31], there was no significant change in color. However, there are some exceptions. Laurita et al. [77] noticed a significant drop in the Luminosity (L), redness (R), and greenness (G) of rocket leaves after PAW treatment, which were similar to changes seen in tomatoes treated with PAW [115]. The degradation of pigments or changes in tissue microstructure can cause these color changes in fresh products. In contrast, Zhao et al. [41] found that PAW treatment could reduce the value of the browning index (BI) in button mushrooms. On the other hand, sanitizers such as ozone and chlorinated water resulted in moderate browning of lettuce washed with them [116].

PAW treatment of meat and fish products saw a significant change in color parameters compared to the agricultural products. PAW used as a curing agent [46,47,117] or directly applied in fresh meat [90] both saw an increase in the redness index (a*), while there were no significant changes in the lightness (L*) or yellowness (b*) indexes. Contrarily, Liao et al. [105] saw a decrease in a* value using PAW as a curing agent. This may be due to the denaturation of the myoglobin’s globin moiety after thawing [118]. Meanwhile, Qian et al. [94] saw no change in L*, a*, or b* values in fresh PAW-treated beef. When applying PAW to chicken meat and skin, the meat part showed a slight decrease in a* values and an increase in L* values, where the skin part showed a slight increase in b* value [76]. A similar result was seen in chicken drumsticks by Qian et al. [104]. As for the fish product, in mackerel fillets, Zhao et al. [91] found a slight reduction in L* and b* values, whereas Liu et al. [78] found an increase in the L* value and a decrease in the a* value in Yellow River carp when treated with PAW.

#### 6.2.2. Texture and Appearance

Texture and appearance have an impact on how the food looks and tastes. It is also a crucial aspect of consumer acceptance. Applying PAW, no significant change in texture, firmness, or appearance was found in the studies on ready-to-eat rocket salad [89], Korean rice cake [32], grapes [70,86], tiger nuts [110], rice [114], and tofu [95]. Moreover, after storage, fresh-cut pears [88] and shiitake mushrooms [42] showed better texture firmness value compared to the control. PAW treatment also marginally delayed the softening of fresh-cut apples [87] and goji berries [108] during storage.

## 7. Hurdle Technology

The most significant benefit of hurdle technology is the capacity to overcome microbial resistance to traditional techniques. Many researchers have already experimented on the combined effect of PAW with other technologies, such as mild heat and ultrasound. Liao et al. [114] found a better, 2.12 log10 CFU/g reduction in *B. cereus* spores in rice when treated by PAW with 55 °C mild heat, which was substantially greater than PAW alone, with a reduction of 0.72 log10 CFU/g. A similar result was found in grapes [86] and salted kimchi cabbage [44]. Combining ultrasound with PAW also showed a promising result. In fresh raw chicken meat [119], *E. coli* and *S. aureus* were reduced by 1.51 log CFU/mL and by 0.85 log CFU/mL, respectively, using the combined approach of PAW and ultrasound, while PAW alone reduced *E. coli* and *S. aureus* by 0.74 log CFU/mL and 0.68 log CFU/mL. This finding perfectly aligns with the recent studies on chicken meat and skin [76] and tomato [120]. Some researchers used other liquids instead of or alongside water to see the changes in the efficacy of PAW. The use of propylparaben (PP) [121], slightly acidic electrolyzed water (SAEW) [105], plasma-activated lactic acid (PALA) [104], and plasma-activated brine (PAB) [47] all showed slight to moderate improvements in disinfection efficacy, but some showed a negative impact on food quality. These results showed a positive sign for combining other treatments with PAW in bacterial inactivation. However, there is not a sufficient amount of data available. Therefore, more research is needed in this regard.

## 8. Challenges and Future Prospective

PAW has shown potential use as a microbial decontaminant in different types of foods. Along with non-thermal treatment, it is also eco-friendly. However, due to its complexity, the whole reaction process is still unclear. Application in real food products has shown slightly lower effectiveness compared with in vitro studies due to the interaction of PAW with other food constituents. The quality of some foods is also affected by different treatment conditions. Sometimes, some substances (for example, organic matter, such as beef extract) interfere the deactivation efficacy of PAW against pathogenic microorganisms [93]. Therefore, knowing the exact antimicrobial mechanism and its reaction with other food constituents is essential for a clear view of the process. It will be a challenge to find the best treatment conditions for different food items without affecting the food quality and safety.

The study of the effect of PAW on food quality is still limited. More work is needed to fully understand the underlying process and to justify the previous results. As PAW contains many reactive elements, future studies should also focus on whether it forms any toxic substances that violate food safety. PAW has been investigated for several years, but as far as we know, recognized authorities such as the World Health Organization (WHO) have not approved it for food applications, and regulatory criteria have not yet been established. We should also focus on developing a low-cost solution for upscaling PAW production before its practical implementation in the food industry.

## 9. Conclusions

In recent years, PAW has received a lot of attention as a decontamination method. Numerous studies have been conducted investigating the effectiveness of PAW in various foods. All the data have shown that PAW could be used as a microbial decontaminant while ensuring food safety and quality. However, the data on quality parameters after PAW treatment are limited. Most of these data showed a positive influence of PAW on food quality, although a few of them also showed some negative effects. The cause of these effects is still unclear and needs to be identified in further studies. Moreover, further studies need to be performed to optimize PAW parameters to best utilize its effectiveness without affecting food quality.

## Figures and Tables

**Table 1:** The impact of plasma-activated water on microorganism disinfection.

Microorganisms	Products	Treatment Conditions	Microbial Reduction	Reference
Methicillin-susceptible *S. aureus* and methicillin-resistant *Staphylococcus aureus*	Cooked chicken	Plasma was activated for 20 min, and the chicken was immersed in PAW for 20 min	2.09 and 2.29 log CFU/g	[40]
Total mesophilic and psychotropic bacteria	Rocket leaves	Leaves were washed with PAW for 2–5 min	1.7–3 log CFU/g	[77]
*Shewanella putrefaciens*	Yellow River carp fillets	Fillets were immersed into PAW for 6 min, which was activated for 120 s	1.03 log CFU/g	[78]
*E. coli* O104	Alfalfa seeds	Seeds were immersed into PAW for 1–16 h	1.67 log CFU/g	[79]
*E. coli* O157	Mung bean seeds	Seeds were immersed into PAW for 1–16 h	1.76 log CFU/g	[79]
*Candida albicans* and *Penicillium Chrysogenum*	Korean rice cake	Samples were treated with PAW for 20 min	~2.0 log CFU/g	[32]
*Salmonella enterica serovar* Enteritidis	Eggs	Eggs were treated with PAW for 0.5 to 2 min	0.77 to 4.41 log CFU/egg	[34]
*Pseudomonas deceptionensis*	Chicken breast	The chicken breast was dipped into PAW for 12 min	1.05 log CFU/g	[35]
Total bacteria	Baby spinach leaves	Leaves were rinsed in PAW with no variability for 8 days at 4 °C	1 log CFU/ml	[80]
*Enterobacter aerogenes*	Grape tomato	Tomatoes were washed with PAW for 3 min	4.65 log CFU/surface	[81]
*S. cerevisiae*	Grapes	Activated water was put into the grape-containing tube after 30 and 60 min exposure with plasma	0.38- to 0.53-log CFU/ml	[73]
Total bacteria	Chinese bayberry	Fruits were soaked in PAW for 0.5 to 5 min and kept for 8 days at 3 °C	1.1 log CFU/g	[39]
Total bacteria	Button mushrooms	Mushrooms were soaked in PAW and stored at 20 °C over 7 days	1.5 log CFU/g	[31]
*Staphylococcus aureus*	Strawberry	Strawberry was immersed into PAW for 5–15 min and kept for 4 days	1.7 to 3.4 log CFU/g	[25]

**Table 2:** The impact of PAW on the quality and shelf life of food.

Products	Treatment Conditions	Impact on Quality Parameters	Shelf Life	Reference
Agricultural Products				
Ready-to-eat rocket salads	PAW was generated with DBD system, and salads were then immersed for 15 min	No change in leaf color or texture was seen	Shelf life was extended from 3 days to 7 days (~2.5 times)	[89]
Tomato	PAW was created by air plasma jet for 1, 3, 5, and 10 min, and the tomato was then soaked in PAW for 15 min	No significant change in color or total phenolic compound		[84]
Baby spinach leaves	Leaves were rinsed with PAW, with no variability for 8 days at 4 °C	No significant change in color	Shelf life was extended	[80]
Button mushroom	500 mL DW was activated for 20 min to form PAW, and mushrooms were then soaked for 5, 10, or 15 min in PAW before being stored at 20 °C for 7 days	No noticeable changes in pH, color, or antioxidant properties, and mushroom softening was prolonged		[31]
Fresh-cut apple	Apple cubes were soaked in PAW for 5 min and kept at 4 °C for 12 days	Reduced superficial browning; no change in firmness, antioxidant content, and radical scavenging activity	Acceptable sensory score was retained longer (12 days) compared to control (6 days)	[87]
Fresh-cut pears	Pear cubes were soaked in PAW for 5 min and kept at 4 °C for 12 days	The amount of soluble solids did not significantly vary; ascorbic acid content and radical scavenging activity were unaffected by storage for 8 days		[88]
Grapes	Water was activated with plasma for 30 and 60 min, and grapes were then soaked in PAW for 30 min	Surface color of grapes did not significantly change, and total anthocyanins demonstrated no significant reduction in cyanidin-3-glucoside equivalents		[73]
Fresh-cut kiwifruit	Water was activated with plasma for 30 min., and PAW was then sprayed on kiwi slice and kept at 4 °C for 8 days	Improved activity of superoxide dismutase, peroxide, and catalase was seen		[96]
Animal-Derived Products				
Mackerel fillets	PAW was activated for 10 min, then for 10 min, the fish cube was immersed in the PAW	No significant change in color		[91]
Yellow River carp fillets	Water was activated with plasma for 120 s, and the fillets were then soaked in PAW for 1.5, 3, 4.5, and 6 min	The L* value of the fillets increased, while the a* value significantly declined, but the b* value remained unchanged compared to control; no change in sensory or textural properties		[78]
Shrimp	Water was activated with plasma for 10 min and then frozen to form ice at −20 °C for 24 h; shrimps were placed on PAW ice and kept at 4 °C for 9 days	The TVBN value was lowered; color and hardness were delayed	Shelf life was extended by 4 to 8 days	[92]
Fresh beef	Water was activated with plasma for 30 min to generate PAW; then, the fresh beef was sprayed with PAW; treatment intervals ranged from 6 to 192 h and stored at 4 °C for 24 days	No noticeable changes in quality parameters when the time interval is larger than or equal to 24 h	Shelf life was extended for 4–6 days	[90]
Cooked chicken surface	PAW was activated for 20 min, and the chicken was then soaked in PAW for 5 min	No change in chicken surface color was seen		[40]
Chicken meat and skin	Water was activated with plasma for 6.5 min to generate PAW, and the chicken meat was then immersed in PAW and ultrasonicated at 4, 25, and 40 °C for 30, 45, and 60 min	Noticeable change in color was seen, but there were no substantial changes in hardness, protein, or fat content		[76]
Eggs	PAW was activated in combination with different power consumption, activation time, and water source, then the egg was soaked in PAW for 30, 60, 90, 120 s	The freshness index was better than the commercial process, and the surface remained relatively intact		[34]
Processed Foods				
Tofu	Water was activated by CAPP for 15 min, and tofu was then immersed in PAW for 30 min or 24 h	The L* values decreased with time, whereas a* and b* values rose; did not affect the springiness, hardness, and gumminess		[95]
Korean rice cake	PAW was generated using distilled water for 20 min with two DBD and used to treat Korean rice cake for 40 min	No noticeable change in color, pH, and firmness was seen		[32]
Thin sheets of bean curd	Water was activated with plasma for 90 s, and pieces of bean curd were then immersed into PAW for 10, 20, and 30 min	No noticeable impact on the overall isoflavone content, sensory qualities, or the majority of textural properties was found		[37]
